# The Influence of Fibers on the Flexural and Tensile Properties of Asphalt Mastic Based on Finite Element Simulation

**DOI:** 10.3390/ma19091882

**Published:** 2026-05-02

**Authors:** Zizhen Li, Kang Zhao, Yidong Chai, Jianfeng Li, Songqiao Yang

**Affiliations:** 1 School of Civil and Architectural Engineering, Liaoning University of Technology, Jinzhou 121001, China; 2 Shanxi Road & Bridge Construction Group Co., Ltd., Taiyuan 030006, China; jianfengli2022@163.com (J.L.);; 3 Shanxi Road and Bridge Renewable Resources Development Co., Ltd., Taiyuan 030006, China; 4 School of Civil Engineering, Taiyuan University of Technology, Taiyuan 030024, China

**Keywords:** asphalt mastic, bending and tensile properties, Burgers’ constitutive model, three-dimensional fiber model

## Abstract

To improve the low-temperature crack resistance of asphalt pavement, this paper investigates the effects of fiber length, content, and type on the flexural and tensile properties of asphalt mastic. Firstly, a numerical program was developed in MATLAB to establish a three-dimensional finite element model of asphalt mastic with an uneven fiber distribution in ABAQUS. Then, the Burgers model selected for simulation was obtained through the asphalt low-temperature bending beam rheological test (BBR). Constructing a three-point bending virtual test of asphalt mastic using a three-dimensional fiber model and systematically analyzing the influence of fiber parameters on bending and tensile properties. The accuracy of the three-dimensional fiber model was verified through BBR experiments. The finite element simulation results show that the addition of fibers can significantly improve the tensile performance of asphalt mastic; increasing the fiber content or length can reduce the peak stress at the bottom of the mid-span and delay cracking. The higher the fiber elastic modulus, the smaller the vertical displacement of the specimen. The model established in this article can effectively elucidate the mechanism of fiber reinforcement, providing a theoretical basis for optimizing fiber parameters and improving the crack-resistance performance of asphalt pavement.

## 1. Introduction

Asphalt pavement, as one of the main forms of modern transportation infrastructure [1], directly affects road service life, driving safety, and maintenance costs due to its performance. In low-temperature environments, asphalt mixtures are prone to brittle cracking [2,3], which seriously restricts the durability of road surfaces in areas with high cold and large temperature differences [4]. To improve the crack resistance of asphalt materials, fiber reinforcement technology is widely used in asphalt mixtures [5,6,7]. Through the bridging, crack-resistance, and reinforcement effects of fibers, the mechanical properties and durability of materials are significantly improved [8]. In this context, fiber-reinforced technology has shown great potential due to its unique mechanisms of “micro reinforcement” and “bridging”. The core function of incorporating discrete short-cut fibers into asphalt materials occurs at the critical scale of asphalt mastic. Asphalt mastic is a uniformly viscous medium formed by mixing asphalt binder and mineral fillers (such as mineral powder) at high temperatures [9], and it plays a crucial role in asphalt mixtures. Not only is it the “glue” that wraps and binds coarse and fine aggregates, but it is also a continuous carrier for stress transmission and distribution. Therefore, the mechanical properties of the adhesive itself, especially its tensile and deformation resistance, fundamentally determine the macroscopic mechanical response and failure mode of the asphalt mixture formed by its bonding.

Adding fibers directly to asphalt mastic means strengthening at the source of stress transmission [10]. Fibers form a three-dimensional, randomly distributed support network within the asphalt mastic matrix [11]. When microcracks occur in the asphalt mastic due to temperature or load stress, fibers can span across both sides of the crack, effectively transmit and redistribute stress, absorb and dissipate energy, thereby delaying or even preventing crack propagation, significantly improving the fracture toughness, tensile strength, and fatigue resistance of the asphalt mastic body [12,13]. At present, there are various types of fibers suitable for asphalt materials, including high modulus mineral fibers (basalt fibers [14], glass fibers [15]), synthetic fibers (polyester fibers [16], polyacrylonitrile fibers [17]), metal fibers [18], and some natural fibers [19]. Their physical and mechanical properties (such as elastic modulus, elongation at break, and surface properties) significantly affect the reinforcement effect and mechanism of the adhesive [20,21,22,23].

Research on fiber-reinforced asphalt materials shows a trend toward deepening from macroscopic performance testing to microscopic mechanism analysis. In recent years, domestic and foreign scholars have conducted experimental and theoretical research on fiber-reinforced asphalt materials [24,25,26]. In terms of experimentation, most studies focus on testing the macroscopic mechanical properties of asphalt materials with added fibers, such as tensile strength, flexural tensile performance, and fatigue characteristics. Through experiments, it was found that the mechanical properties of asphalt materials with added fibers were significantly improved. In numerical simulation, especially the finite element method, it has become an indispensable tool for exploring the micromechanical behavior of composite materials, as it can reveal the invisible stress and strain fields within the material and enable low-cost, efficient virtual experiments on design parameters [27]. Previous studies have analyzed the influence of fiber distribution and orientation, and their interaction with the matrix, on material properties using two-dimensional or simplified three-dimensional models. However, most existing numerical models are based on idealized assumptions about fiber distribution, such as uniform or regular directional arrangements [28]. This is far from the true dispersion state of random, disordered, and interlaced fibers formed after high-speed shear mixing in the actual preparation process of asphalt mastic. This simplification significantly affects the model’s prediction of stress transfer, stress concentrations, and final failure modes at the fiber–matrix interface. More importantly, as a typical viscoelastic material, asphalt mastic exhibits significant time and temperature dependence in its mechanical properties. In low-temperature environments, creep- and stress-relaxation behavior are the primary factors determining crack resistance. Therefore, a numerical model that can accurately predict the low-temperature performance of fiber-reinforced asphalt mastic must be based on a precise viscoelastic constitutive description of the asphalt mastic itself.

In summary, existing research still has the following shortcomings: (1) most models assume uniform distribution of fibers, which fails to truly reflect the random distribution characteristics of fibers in actual construction; (2) lack of simulation research on the influence of different fiber lengths, contents, and types in asphalt mastic; (3) the viscoelastic constitutive behavior of the fiber asphalt mastic system under low temperature conditions and its influence mechanism on bending and tensile properties are not yet clear. To fill the research gap mentioned above, this paper is based on rheological testing (BBR) of asphalt low-temperature bending beams to obtain the parameters of the Burgers viscoelastic constitutive model for the asphalt binder. Using MATLAB and ABAQUS, a three-dimensional microscopic finite element model that considers random fiber distribution is established to systematically simulate the mechanical response of asphalt mastic under three-point bending loading. The aim of this study is to elucidate the synergistic effects of fiber length, content, and type on the flexural and tensile properties of asphalt mastic and to verify the model’s reliability through BBR experiments. Based on the rheological testing (BBR) of asphalt and the numerical modeling approach described above, this article aims to elucidate the mechanical mechanism of fiber-reinforced asphalt mastic at the microscale, providing scientific support for optimizing fiber parameters and for understanding the low-temperature cracking mechanism of asphalt materials.

## 2. Materials and Methods

### 2.1. Preparation of Test Materials and Samples

To obtain the material properties of an asphalt mixture at a microscopic level, asphalt mastic is used to characterize the material properties of the asphalt mixture except for aggregates and voids [29,30]. This study selected Qinhuangdao 70 # matrix asphalt (70 # is the grade of asphalt binder, determined by the degree of penetration) as the asphalt binder, and its main physical properties were reported in the author’s previous research [31]. The preparation of asphalt mastic involves adding mineral powder to the base asphalt at a 1:1 weight ratio. Asphalt mastic is produced according to a procedure to obtain a homogeneous asphalt filler mixture. The program consists of four basic steps [31]: 1. Firstly, the packing is dried in a 150 °C oven for 17 h. Then store it in a vacuum dryer until it cools to room temperature. 2. Heat the asphalt and filler in a 150 °C oven for 1 h. 3. Use a mechanical mixer that rotates at 2400 revolutions per minute to mix asphalt and filler. Slowly add the filler to the asphalt for 20 min to avoid particle settling. 4. Finally, let the asphalt filler mixture cool and store it in the refrigerator.

### 2.2. Low Temperature Asphalt Binder Bending Beam Rheological Test (BBR)

**Figure 1:**
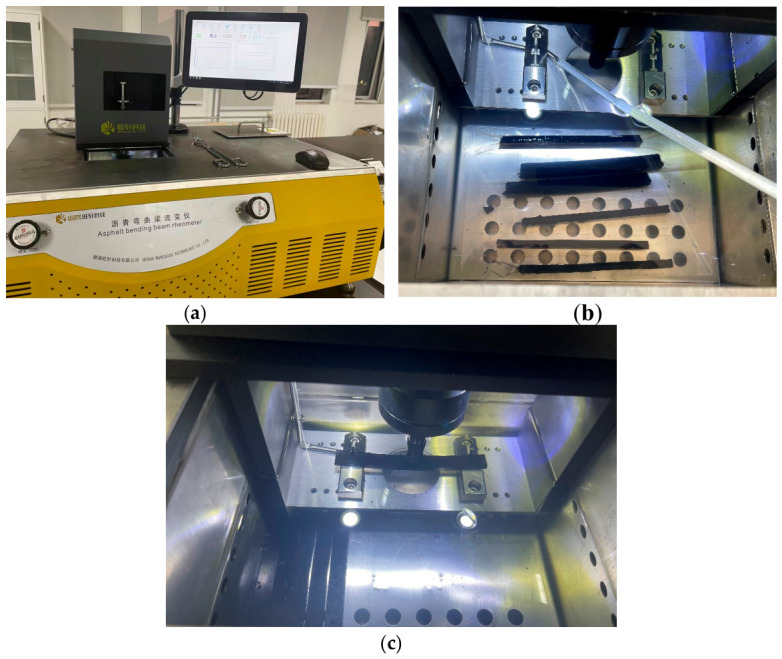
Test setup diagram (Haoxuan, Hunan, China). (**a**) The diagram of the bending beam rheometer equipment; (**b**) BBR small beam insulation treatment; (**c**) Experimental loading process.

The asphalt bending creep stiffness test in this study was conducted on a TE-BBR bending beam rheometer, and the instrument and test process are shown in Figure 1. Figure 1a is the diagram of the bending beam rheometer equipment, Figure 1b is the insulation treatment of the small beam in the equipment to reach the test temperature, and Figure 1c shows the loading of the equipment.

**Figure 2:**
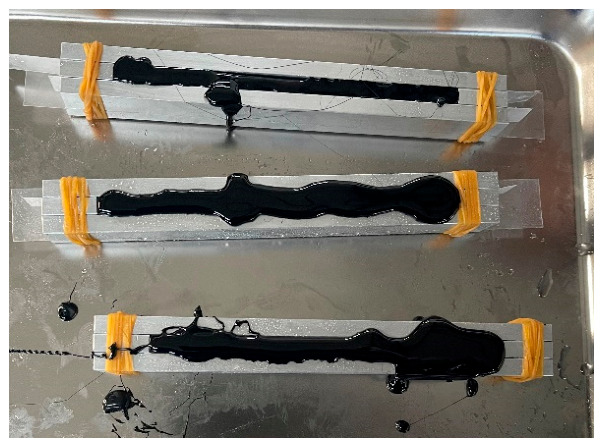
Schematic diagram of the BBR specimen before demolding.

The preparation steps for the bending beam rheological test samples are as follows: (1) Heat the asphalt sample in an oven to a flowing state, with a heating temperature of 135 °C. (2) Apply a mixture of propylene glycol and talc powder on the inside of the test mold, then place a plastic sheet and tie it with a rubber band. (3) Pour the flowing asphalt back and forth into the mold from one end to the other, making the asphalt slightly higher than the mold. Cool the mold filled with asphalt at room temperature for 1 h and then use a hot knife to scrape off any excess asphalt on the surface. (4) Demold the scraped small beam specimens for bending beam rheological testing. The sample preparation is shown in Figure 2. The purity of the Qinhuangdao 70 # base asphalt in this study is >99.5% (as determined by the solubility test). Mineral filler (limestone powder) with a particle size of <0.075 mm (through 200 mesh) and a CaCO_3_ purity of >98%. Fine particle size ensures good dispersibility and increases the specific surface area, thereby improving the interaction between asphalt and filler. Talc (mold release agent) is an industrial-grade powder with an average particle size of 10–15 μm, used to prevent asphalt from adhering to molds. The particle size range provides a uniform coating without affecting the sample surface.

Asphalt binder exhibits significant creep characteristics under external forces at low temperatures. By studying the creep performance of asphalt under external forces, the suitability of asphalt pavement for low-temperature environments can be assessed. The SHRP program in the United States uses a bending beam rheometer to measure the low-temperature creep stiffness of asphalt binders in the research process of asphalt PG grading. The bending beam rheological test, abbreviated as BBR test, is used to determine the low-temperature creep characteristics of asphalt binders by measuring flexural deformation under constant load at low temperatures. The size of the small beam specimen in the BBR test (length × width × height) is 127 mm × 6.35 mm × 12.7 mm. In the test, a load of 0.980 N is applied to the small beam for 240 s, and the deflection of the small beam specimen is measured using the displacement sensor in the system. After 240 s, the load is removed, and the test is stopped. The low-temperature performance of asphalt is evaluated by the stiffness modulus S and creep rate m, and the S and m values are calculated using Equations (1) and (2) [32].

According to the principle of elasticity viscoelasticity correspondence, the stiffness modulus of linear viscoelastic materials can be assumed. When a linear viscoelastic beam is subjected to a constant load at time t = 0, and the load remains constant, the stress distribution inside it is exactly the same as that of a linear elastic beam subjected to the same load. However, strain and displacement will vary over time and can be obtained by replacing the elastic modulus E in the elastic solution with 1/D(t). Since 1/D(t) is equivalent to S(t), after reorganizing the elastic solution, the following stiffness relationship is obtained:

(1) $$S\left(t\right)=\frac{PL^{3}}{4bh^{3}\delta \left(t\right)},$$

(2) $$m\left(t\right)=\left|\frac{d\mathrm{lg}S\left(t\right)}{d\mathrm{lg}t}\right|,$$

In the formula: S(t) is the bending creep stiffness that varies with time, MPa; P = load, N; L = span, mm; b = width of beam, mm; h = thickness of beam, mm; $\delta \left(t\right)$ = deflection of beam, mm.

### 2.3. Numerical Simulation Study on Fiber-Containing Asphalt Mastic

#### 2.3.1. Generation of 3D Numerical Models

The finite element (FE) simulation used in this article is divided into two terms: the asphalt matrix term and the fiber term. The Weibull distribution is used in the model to describe fiber distribution. Therefore, FE models can simultaneously consider different degrees of fiber dispersion and fiber distribution forms. Based on previous research [33,34], a generation algorithm for the three-dimensional random distribution of different fiber types within the rectangular asphalt binder matrix is proposed. Finally, using this algorithm, MATLAB (MATLAB R2023b) was used to generate an inp file for import into ABAQUS (ABAQUS 2023), and a three-dimensional finite element analysis model was established in ABAQUS.

**Figure 3:**
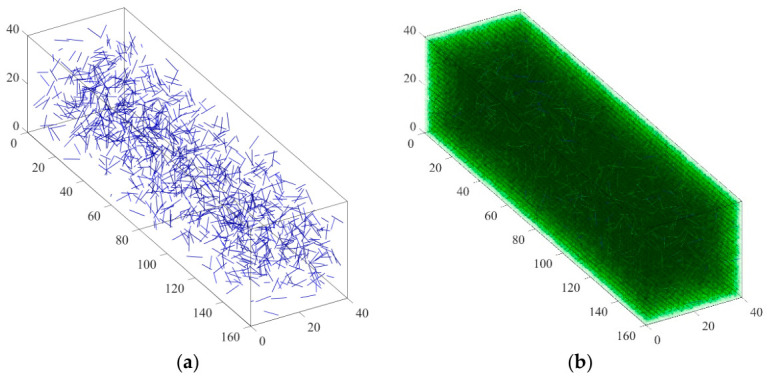
3D model of randomly distributed fibers. (**a**) MATLAB generates models with dimensions (160 mm × 40 mm × 40 mm); (**b**) finite element mesh division model.

In random number generation, the recursive formula Xn + 1 = R (X1, X2, …, Xn) is commonly used to generate a sequence, where R is a recursive function. The random number sequence generated by this method is completely determined by the initial value and the recursive formula and, therefore, cannot fully meet the strict requirements for statistical randomness and independence. It is generally referred to as a “pseudo-random number sequence”. However, even when the number of generated sequences is small, such sequences can still simulate real random phenomena well. Meanwhile, due to the fixed initial conditions and recursive rules, time series can be repeatedly generated, which is convenient in practice. Based on the above principles, the rand () function built into MATLAB is used directly in subsequent simulations to generate the required pseudo-random numbers, which are then used for numerical modeling of fiber position and direction (represented by their endpoints). Figure 3 shows the random distribution of fibers generated in the rectangular specimen model of asphalt mastic.

#### 2.3.2. Numerical Simulation Constitutive Model

Asphalt binder is a viscoelastic material, exhibiting both viscous and elastic flow characteristics. In the mechanical analysis of viscoelastic materials, materials exhibit two types of mechanical properties: linear viscoelasticity and nonlinear viscoelasticity. Research has shown that, within a certain time range, the linear viscoelastic properties of viscoelastic materials can be represented by various models composed of Hooke’s law springs and Newton’s law clay pots, and material parameters do not change with increases or decreases in stress and strain. For materials with nonlinear viscoelastic properties, simple model combinations cannot be used, and their mechanical properties and constitutive relationships will be more complex [35].

**Figure 4:**
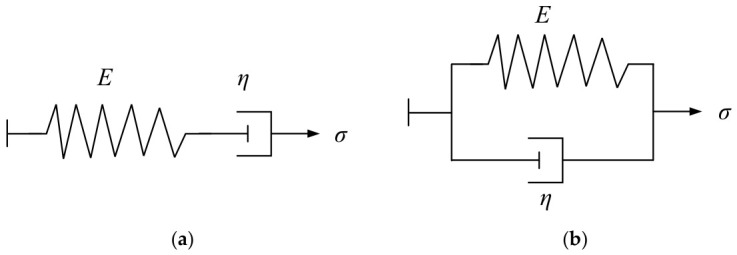
The structure of two constitutive equations. (**a**) Maxwell model; (**b**) Kelvin model.

Relaxation and creep are typical viscoelastic behaviors of solids, and viscoelastic models are usually used to describe them. The viscoelastic model is obtained by connecting basic physical components in various series and parallel configurations, among which the most classic models are the Maxwell and Kelvin models. The Maxwell model consists of elastic and viscous elements connected in series, while the Kelvin model consists of elastic and viscous elements connected in parallel. The structures of the two models are shown in Figure 4.

**Figure 5:**
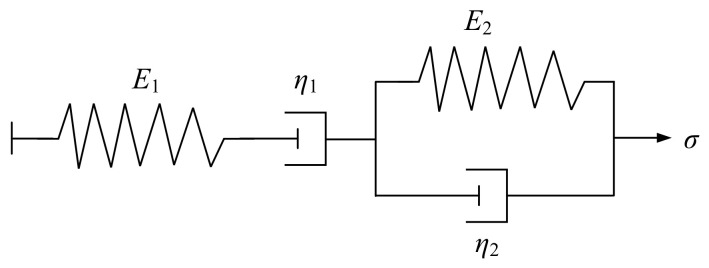
Burgers model structure diagram.

The most commonly used model for studying the rheological properties of asphalt binders is the Burgers model. The Burgers model is composed of the Maxwell and Kelvin models in series and is part of the four-element model. It can well describe the mechanical behavior of asphalt binder within a certain temperature range and determine the constitutive equation by solving for the viscoelastic parameters of the mechanical components, thereby predicting the material’s stress–strain relationship [36,37,38]. The structure of the Burgers model is shown in Figure 5 [39], and the constitutive equation is shown in Equation (3) [40].

(3) $$\sigma +p_{1}\dot{\sigma }+p_{2}\ddot{\sigma }=q_{1}\dot{\varepsilon }+q_{2}\ddot{\varepsilon },$$

where $p_{1}=\left(\eta_{1}E_{1}+\eta_{1}E_{2}+\eta_{2}E_{1}\right)/E_{1}E_{2}$, $p_{2}=\eta_{1}\eta_{2}/E_{1}E_{2}$, $q_{1}=\eta_{1}$, $q_{2}=\eta_{1}\eta_{2}/E_{2}$.

By introducing a certain stress input $\sigma =\Delta \left(t\right)\times \sigma_{0}$ into the constitutive Equation (3) and using a series of mathematical deductions, such as the Laplace transform, the Burgers model creep equation can be obtained as [37]:

(4) $$\varepsilon \left(t\right)=\sigma \left[\frac{1}{E_{1}}+\frac{1}{\eta_{1}}t+\frac{1}{E_{2}}\left(1-e^{-\frac{E_{2}}{\eta_{2}}t}\right)\right],$$

where $\varepsilon \left(t\right)$ is the strain at time t; σ is stress; t is the loading time; E1 and $\eta_{1}$ are the elastic and damping coefficients of the Maxwell model, respectively; E2 and $\eta_{2}$ are the elastic and damping coefficients in the Kelvin model, respectively.

Implementation of the Burgers Model in ABAQUS

In ABAQUS, the mechanical behavior of viscoelastic materials is characterized by Prony series form relaxation modulus parameters, including time and frequency domain-related types. This study uses the former to describe the viscoelastic properties in terms of shear relaxation modulus, and the expression for the Prony series is:

(5) $$G\left(t\right)=G_{\infty }+\sum_{i=1}^{n}G_{i}e^{-\frac{t}{\tau_{i}}},$$

Among them, G and Gi are the shear moduli, and $\tau_{i}$ is the shear relaxation time of the series-connected components. The specific conversion process of the Burgers model is as follows:

Convert the shear modulus and elastic modulus using Equations (6) and (7).

(6) $$G_{1}=\frac{E_{1}}{2\left(1+\mu \right)},$$

(7) $$G_{2}=\frac{E_{2}}{2\left(1+\mu \right)},$$

Perform the Laplace transform on the Burgers model constitutive equation to obtain the relaxation modulus, as shown in Equation (8):

(8) $$\bar{Y}\left(s\right)=\frac{q_{1}s+q_{2}s^{2}}{s\left(1+p_{1}s+p_{2}s^{2}\right)},$$

Further transformation can be achieved through the Laplace inverse transform in the time domain, as shown in Equation (9):

(9) $$Y\left(t\right)=\frac{q_{2}}{p_{2}\left(\alpha -\beta \right)}\left[\left(\alpha -\frac{q_{1}}{q_{2}}\right)e^{-\alpha t}+\left(\frac{q_{1}}{q_{2}}-\beta \right)e^{-\beta t}\right],$$

Among them, the shear modulus is:

(10) $$G\left(t\right)=0.5Y\left(t\right)=\frac{G_{1}}{\alpha -\beta }\left[\left(\frac{G_{2}}{\eta_{2}}-\beta \right)e^{-\beta t}-\left(\frac{G_{2}}{\eta_{2}}-\beta \right)e^{-\alpha t}\right],$$

Standardized processing:

(11) $$G\left(t\right)=G_{\infty }+G_{0}\left(g_{1}e^{-\frac{t}{\tau_{1}}}+g_{2}e^{-\frac{t}{\tau_{2}}}\right),$$

where $G_{\infty }=0$, $G_{0}=G_{1}$, $g_{1}=\frac{1}{\alpha -\beta }\left(\frac{G_{2}}{\eta_{2}}-\beta \right)$, $g_{2}=\frac{1}{\beta -\alpha }\left(\frac{G_{2}}{\eta_{2}}-\alpha \right)$, $\tau_{1}=\frac{1}{\beta }$, $\tau_{2}=\frac{1}{\alpha }$, $\alpha =\frac{1}{2p_{2}}\left(p_{1}+\sqrt{p_{1}^{2}-4p_{2}}\right)$, $\beta =\frac{1}{2p_{2}}\left(p_{1}-\sqrt{p_{1}^{2}-4p_{2}}\right)$.

By using the above conversion method, fitting the strain time curve and converting it into the Prony series form of shear relaxation modulus, the four parameters of the Burgers model can be obtained. Implemented g_1_, g_2_, in ABAQUS; $\tau_{1}$, $\tau_{2}$ is the parameter of the viscoelastic material, g_i_ is the shear coefficient of the relaxation modulus and $\tau_{i}$ is the relaxation time [39].

Meanwhile, in this study, we also conducted numerical experiments to investigate the effects of different fiber types on asphalt binder performance. The following fibers were studied: basalt fiber (BF), steel wool fiber (SWF), polyester fiber (JZXW), glass fiber (BLXW), and bamboo fiber (ZXW). Their mechanical properties are shown in Table 1.

#### 2.3.3. Numerical Simulation of Low-Temperature BBR

We studied the mechanical properties of asphalt binder at low temperatures using BBR experiments and, as a reinforcing material, examined how the content, type, and length of fibers affect the mechanical properties of asphalt materials. However, given the difficulty of designing and constructing internal fiber structures in macroscopic experiments, we conducted numerical simulations. Using ABAQUS to simulate the low-temperature BBR test of asphalt binder. The dimensions of the beam specimen (length × width × height) are set to 160 mm × 40 mm × 40 mm.

**Figure 6:**
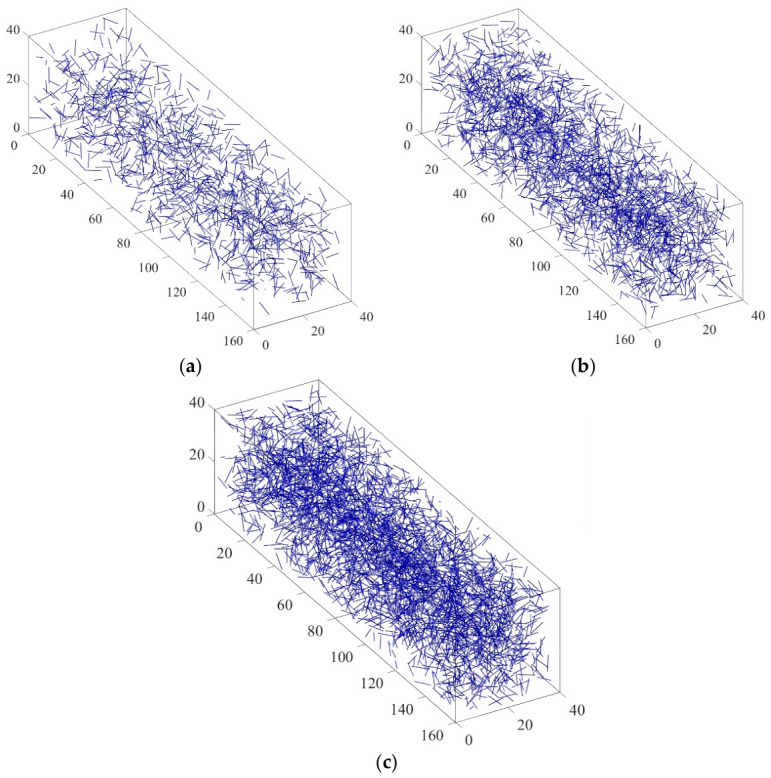
Asphalt mastic models with different fiber contents: (**a**) 0.1% fiber content; (**b**) 0.2% fiber content; (**c**) 0.3% fiber content.

**Figure 7:**
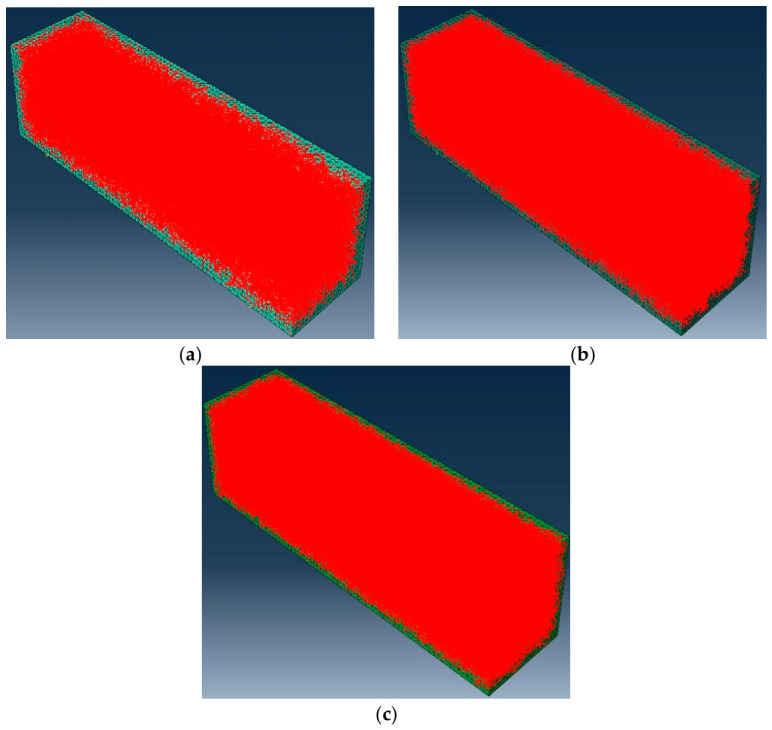
A 3D finite element analysis model: (**a**) 0.1% fiber content; (**b**) 0.2% fiber content; (**c**) 0.3% fiber content.

The fiber content in the asphalt binder in this study is expressed as the weight of the asphalt binder. Before conducting numerical experiments, it is necessary to determine the total number of fibers in the model. This paper determines the number of fibers by calculating the ratio of the total fiber weight to the weight of an individual fiber. When the weight of asphalt mastic is 0.1% and 0.2%, the corresponding total fiber weight is 1.6 g and 3.2 g. Given a fiber length of 6 mm, a diameter of 0.02 mm, and a density of 2.7 g/cm^3^, the weight of a fiber can be calculated. Figure 6 shows three-dimensional random models of BBR samples with different fiber contents generated in MATLAB. In the following figures and labels, a uniform naming convention is adopted: the first number indicates fiber length (mm), the second number indicates fiber content (%), and the letters indicate fiber type (BF, SWF, JZXW, BLXW, or ZXW; see Table 1). For example, “10-02ZXW” denotes a fiber length of 10 mm, a fiber content of 0.2%, and bamboo fiber (ZXW). Figure 6 and Figure 7 show the three-dimensional finite element models of BBR specimens generated in ABAQUS. In the virtual specimen of the virtual experiment, the asphalt binder matrix is modeled as a three-dimensional solid element, and the fibers are modeled as truss elements. The unit types of matrix and fiber are C3D8 and T3D2, respectively. In this study, it is assumed that there is perfect adhesion between the matrix and fiber.

**Figure 8:**
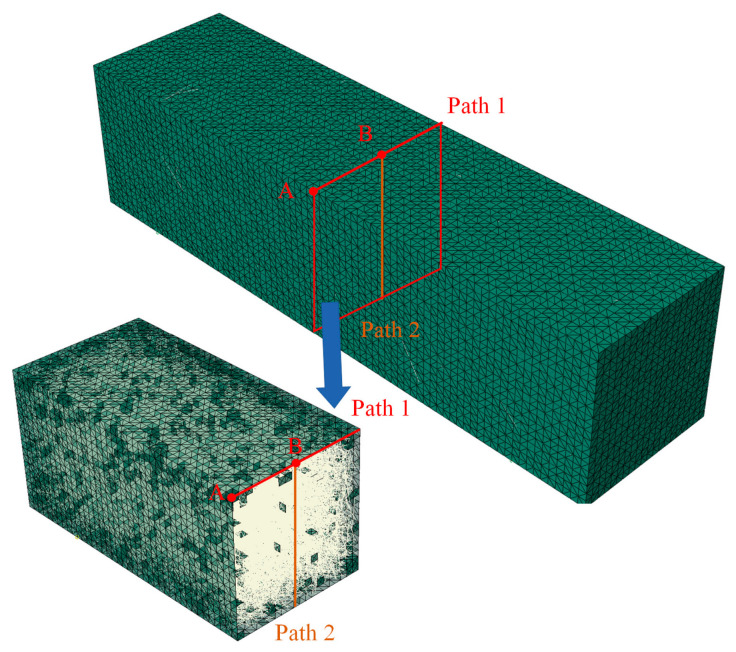
Simulated observation points and observation paths.

To illustrate the influence of fibers on BBR samples in this study, different observation points and paths were simulated to analyze the simulation results, mainly consisting of two nodes and two paths, as shown in Figure 8.

## 3. Results

### 3.1. BBR Test Results and Analysis

**Figure 9:**
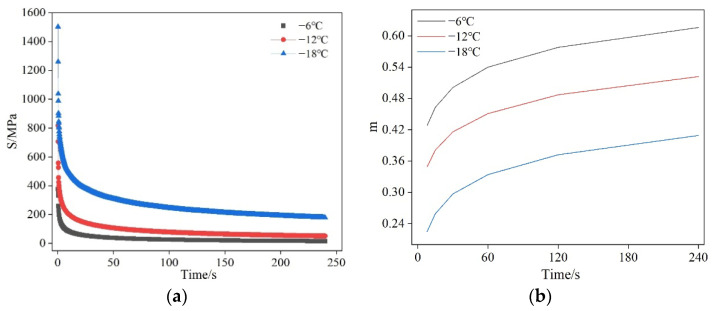
Time-varying curves of stiffness modulus and creep rate of asphalt binder at different temperatures. (**a**) Stiffness modulus; (**b**) m value.

**Figure 10:**
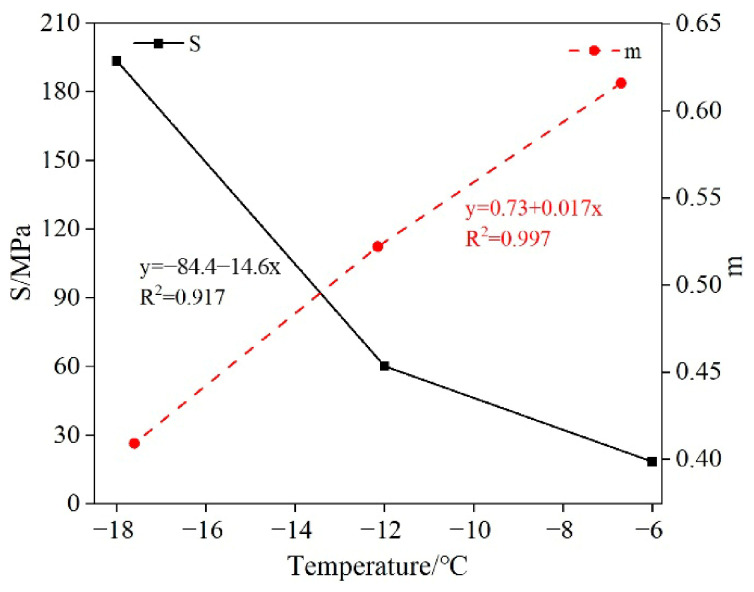
Stiffness modulus, temperature curve, and creep rate temperature curve.

Low-temperature bending beam rheological tests were conducted on asphalt binders at temperatures of −6 °C, −12 °C, and −18 °C. While high-temperature rutting resistance is also critical for asphalt pavements, this study focuses exclusively on low-temperature cracking behavior. The selected test temperatures (−6, −12, −18 °C) follow standard BBR testing protocols for cold-region applications. The test samples were the original 70 # base asphalt samples. The variation in the stiffness modulus and creep rate of asphalt binder with time at different temperatures is shown in Figure 9. Meanwhile, because the temperature dependencies of the stiffness modulus and creep rate of asphalt binder differ, this study linearly fitted the stiffness modulus temperature curve to the creep rate temperature curve. The fitting results are shown in Figure 10.

It can be seen from Figure 9 that: with the increase in loading time, the stiffness modulus of asphalt rapidly decays in the initial period, then the decay rate gradually decreases, and then the growth rate tends to stabilize. As shown in Figure 10, the stiffness modulus of asphalt binder decreases continuously with increasing temperature, while the creep rate increases continuously with increasing temperature.

### 3.2. Determination of Burgers Model Parameters

**Figure 11:**
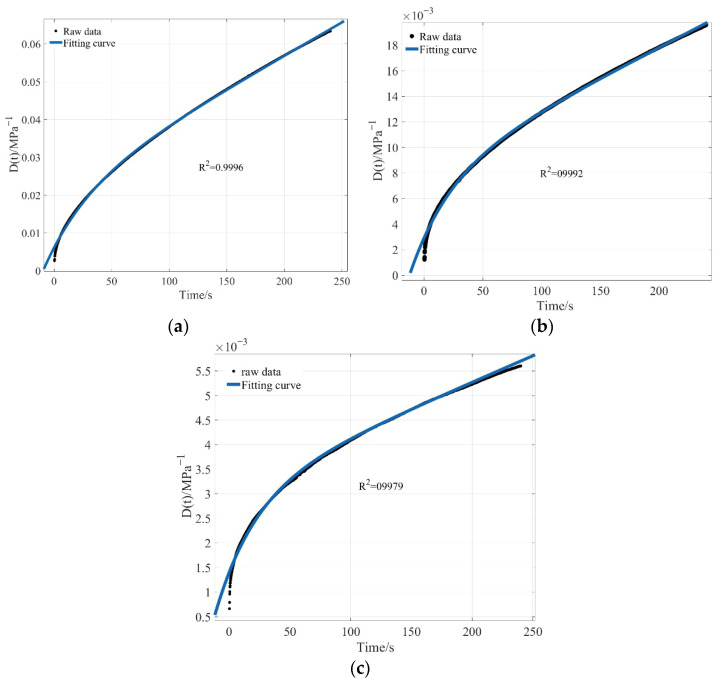
Fitting results of experimental data at different temperatures: (**a**) −6 °C; (**b**) −12 °C; (**c**) −18 °C.

Based on the Burgers model presented in Section 2.3.2, the fitting process is described using the experimental data of 70 # original asphalt at different temperatures as an example. The experimental creep compliance loading-time data were entered into MATLAB and, together with the characteristics of the Burgers model, used to develop a non-linear curve-fitting function. The fitting results are shown in Figure 11.

From Figure 11, the Burgers model fits the asphalt creep compliance loading-time curve well, with correlation coefficients greater than 0.99. The fitting results of the viscoelastic parameters of 70 # matrix asphalt at three temperatures are shown in Table 2. In the process of fitting the creep compliance loading time curve, the fitting error at the beginning of the curve is relatively large, while the fitting error at the middle and end of the curve is relatively small, indicating that the Burgers model can accurately describe and analyze the creep behavior during asphalt BBR testing.

**Figure 12:**
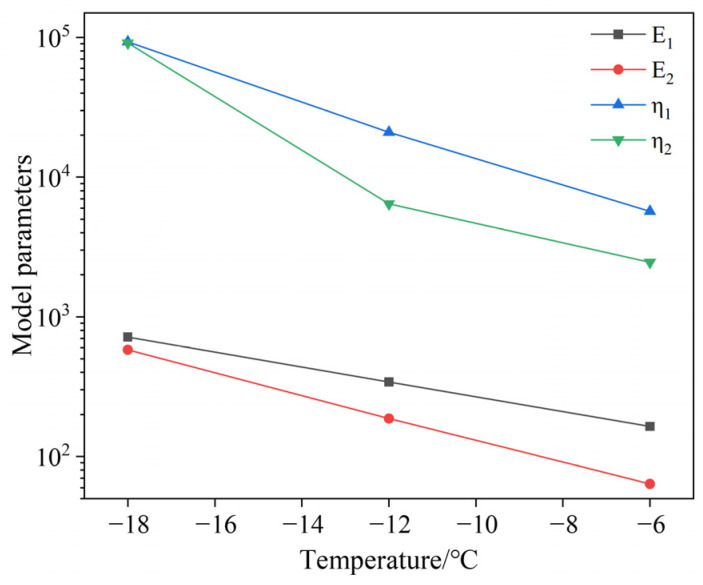
The variation in Burger model parameters with temperature.

According to the fitting results in Table 2, plot the variation in low-temperature viscoelastic parameters with temperature, as shown in Figure 12.

From Table 2 and Figure 12, it can be observed that the viscosity flow parameter $\eta_{1}$, the instantaneous elasticity parameter E_1_, the delayed viscosity parameter $\eta_{2}$, and the delayed elasticity parameter E_2_, which reflects the viscoelastic characteristics of asphalt in the Burgers model, exhibit different patterns of variation. From the perspective of the proportion of viscoelastic parameters, the proportion of $\eta_{1}$ and $\eta_{2}$ is much greater than that of E_1_ and E_2_, indicating that in the Burgers model composed of Maxwell and Kelvin models, the parameters that represent the viscosity characteristics of asphalt are greater than those that represent the elasticity characteristics. Compared to the values of E_1_ and E_2_, the values of $\eta_{1}$ and $\eta_{2}$ are generally larger, typically by one to two orders of magnitude. From the perspective of changes in viscoelastic parameters with temperature, as the test temperature increases, the $\eta_{1}$, $\eta_{2}$, E_1_, and E_2_ of 70 # matrix asphalt all decrease, and the decrease in E_1_ is smaller than that in $\eta_{1}$, $\eta_{2}$, and E2. According to the calculation equation in Section 2.3.2, obtain the viscoelastic parameters of the Burgers model for asphalt binder at low temperatures, convert them into Prony series, and write them into the ABAQUS software. The specific values are shown in Table 3.

### 3.3. Model Validation

**Figure 13:**
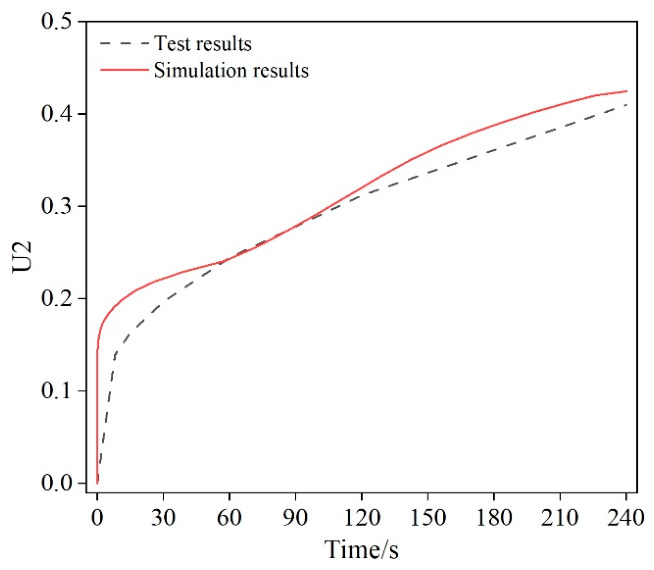
Comparison between numerical simulation results and indoor test results.

The virtual specimen in the virtual experiment consists of asphalt binder beams and rigid bodies that apply loads to the specimen. The asphalt binder matrix is a small beam that is 160 mm long, 40 mm wide, and 40 mm high. The midspan was compared between the BBR small-beam test and the virtual test, and the model’s accuracy was evaluated. Compare the numerical simulation results with the indoor experimental results to verify the model’s accuracy. The experimental and simulated results are shown in Figure 13. From Figure 13, the displacement curves of the virtual and actual experiments are generally consistent, indicating that the simulation method is in good agreement with the actual situation and that the model is relatively accurate. Relevant analysis and research can be conducted based on this. Figure 13 shows that, during rheological deformation, the simulation results are generally consistent with the experimental results. Therefore, the experimental results can demonstrate the effectiveness and regularity of the simulation. In addition, the method of calculating the relaxation modulus using the Prony series is both correct and feasible, enabling accurate characterization of the viscoelastic behavior of the asphalt mastic matrix and its incorporation into ABAQUS. Based on this model and constitutive equation, the synergistic effects of fiber length, content, and type on the flexural and tensile properties of asphalt mastic can be further analyzed.

### 3.4. Effects of Different Fiber Lengths and Contents

**Figure 14:**
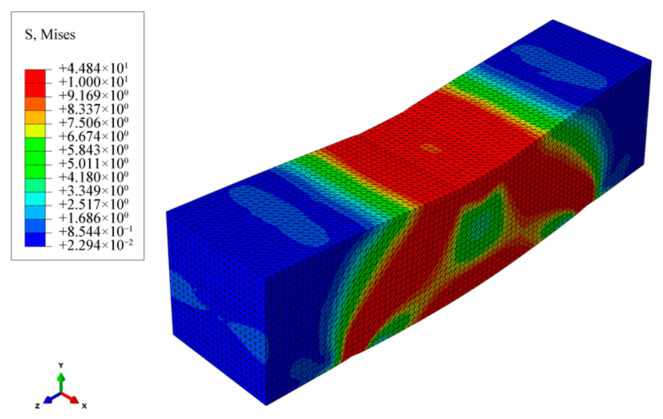
Stress distribution cloud map of asphalt mastic specimens.

**Figure 15:**
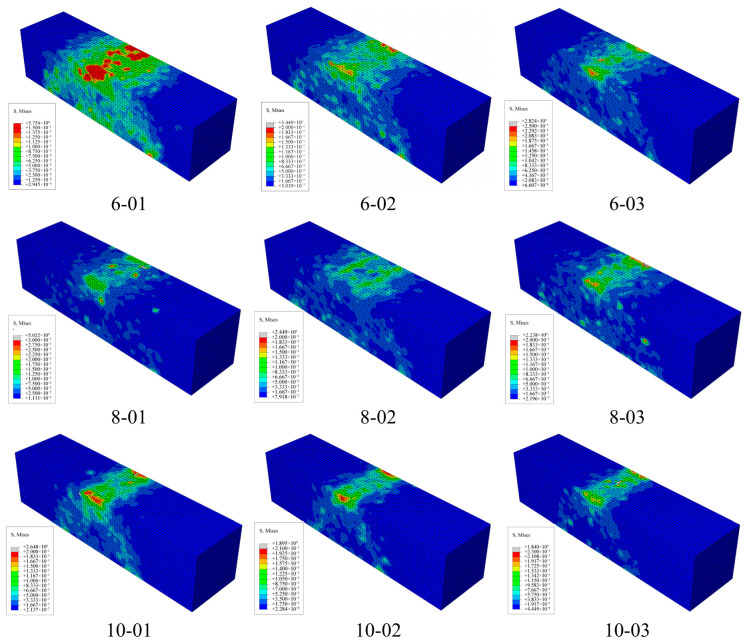
Stress distribution cloud map of asphalt mastic specimens with different fiber contents.

**Figure 16:**
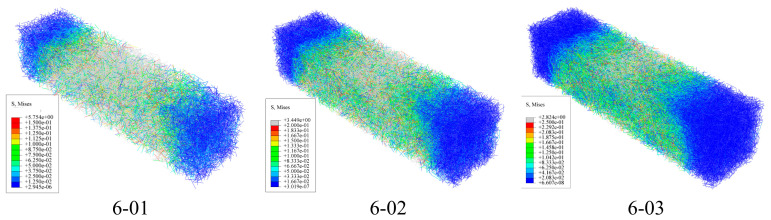

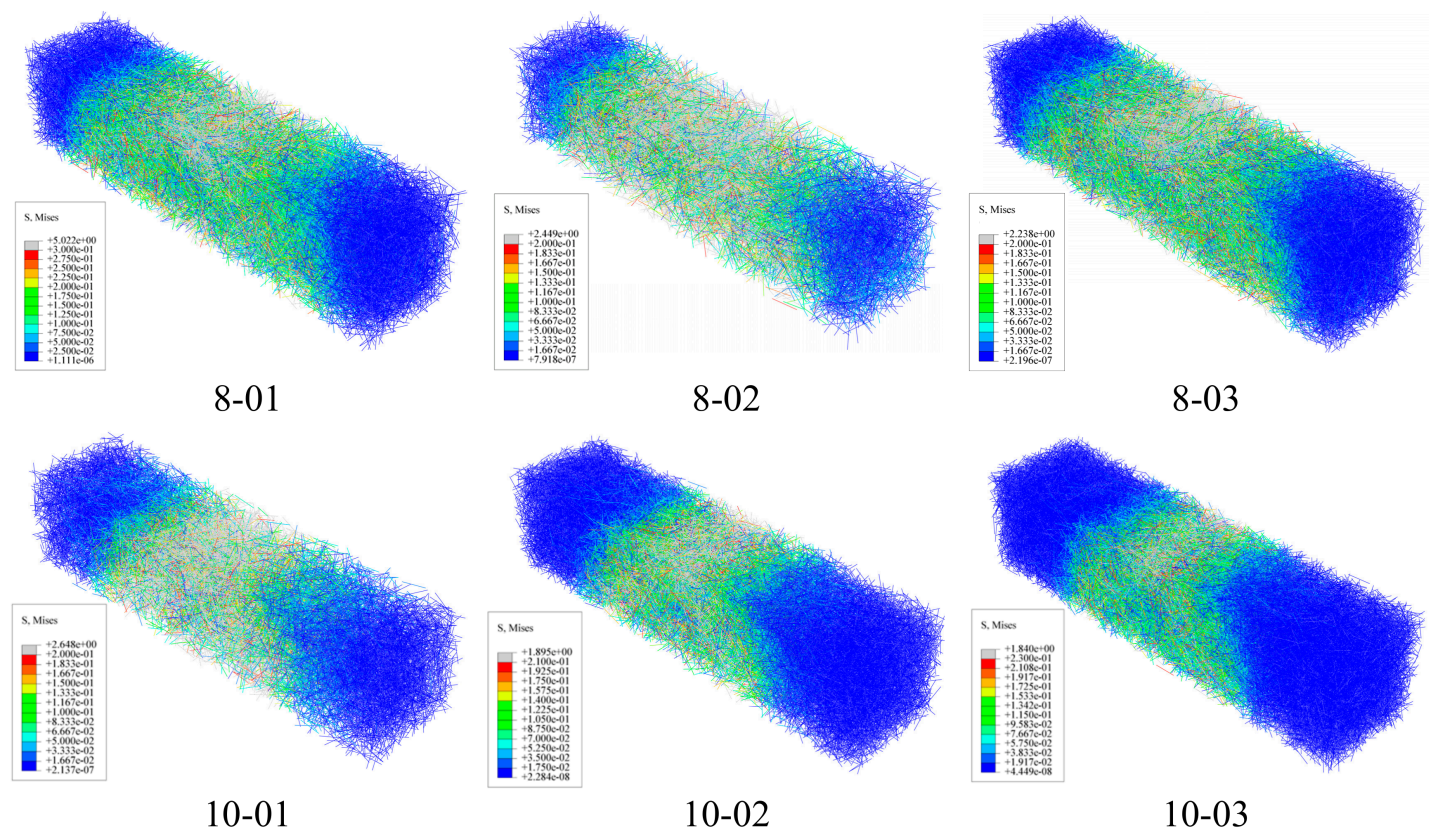
Stress distribution cloud map of fibers in asphalt mastic specimens containing different fibers.

Based on a 3D microstructure model, the stress distribution of beam samples with different contents and lengths of BFs was simulated. Figure 14 shows the stress distribution cloud map of asphalt mastic specimens, while Figure 15 and Figure 16 show the stress distribution cloud maps of asphalt mastic specimens with different fiber contents and their internal fibers. The stress distribution of asphalt beams in the X direction is stepped, with compression at the top and tension at the bottom. The tensile stress is highest at the midspan and bottom of the beam, where the beam is prone to rupture. From the stress cloud map of the midspan section, it can be seen that the tensile stress of the fiber is relatively high, but it plays a reinforcing role in the asphalt binder. Our future work will include actual three-point bending tests on fiber-reinforced asphalt mastic specimens to provide a more comprehensive validation of the numerical predictions.

At the same load displacement, the peak tensile stress decreases with increasing fiber bundle length. When the fiber length increases, its reinforcement effect becomes more significant. The peak tensile stress of basalt fiber is the smallest at a size of 10mm, and the tensile stress gradually increases as the fiber size decreases. This is mainly because the tensile strength of fibers is much higher than that of asphalt binders, and fibers bear more tensile stress, thereby reducing the peak stress in the asphalt binder matrix. Therefore, longer fiber sizes have a better reinforcing effect on asphalt binders. Meanwhile, the figure shows that as the fiber content in the specimen increases, the peak stress of the asphalt binder gradually decreases, and the effect of fiber content in the asphalt mastic on peak stress is more pronounced. From the stress cloud map distribution calculated in Figure 14, Figure 15 and Figure 16, it can be seen that the fibers in the asphalt mastic can absorb the stress in the sample matrix, causing the redistribution of internal stress and, to some extent, reducing the compressive stress values inside the matrix and at the top of the sample.

Therefore, after adding fibers to the pure asphalt mastic specimens, the deformation of the asphalt mastic decreases. Fibers can have a positive reinforcing effect on asphalt mastic in fiber composite materials. At 1800s, as the fiber content in the sample increases, the stress field of the asphalt mastic matrix gradually decreases, and the vertical displacement at the top of the specimen also decreases. The specific results are shown in Table 4. According to Table 4, as the fiber content and length increase, the compressive stress values of the asphalt mastic model decrease compared to the control model. The effect of fiber content becomes more pronounced as the fiber length decreases. When the fiber content is low, the effect of fiber length is also more significant. Adding fibers can improve the compression resistance of asphalt mastic.

**Figure 17:**
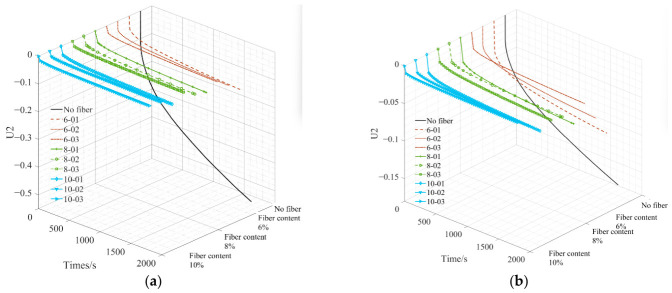
The variation in displacement of points A and B in the specimen with loading time. (**a**) Point A; (**b**) point B.

Figure 17 shows the displacement of points A and B in the asphalt mastic specimen as a function of loading time. Figure 17 shows that adding fibers to the asphalt mastic significantly reduces the vertical displacement at the top of the specimen, indicating improved material properties. As the fiber length increases in the asphalt mastic, the vertical displacement value also gradually decreases. The specific reduction values at points A and B are shown in Table 5. According to Table 5, as the fiber content and length increase, the vertical displacement of the asphalt mastic model decreases compared to the control model. Under the same fiber content, the longer the fiber length, the greater the reduction in vertical displacement. When the fiber length is small, the influence of fiber content on vertical displacement is more significant. The vertical displacement value at point A is greater than that at point B, but the rate of decrease in vertical displacement at point A is significantly higher than that at point B. This indicates that adding fibers to the asphalt mastic matrix can effectively improve the composite material’s overall mechanical properties and help prevent damage.

**Figure 18:**
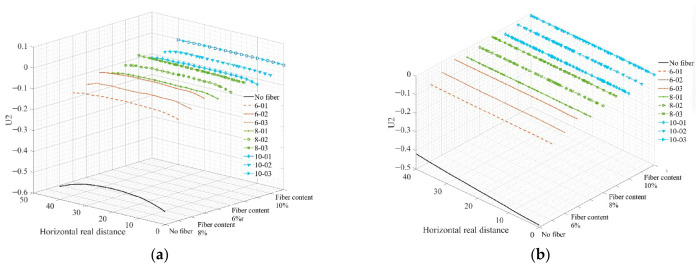
Changes in displacement of path 1 and path 2 along the true position of the specimen. (**a**) Path 1; (**b**) path 2.

Figure 18 shows the variation in the displacements of paths 1 and 2 along the real position of the virtual specimen. According to Figure 18, the displacement decreases significantly after adding fibers in both paths. Meanwhile, the displacement of path 1 is also smaller than that of path 2.

### 3.5. Effects of Different Fiber Types

**Figure 19:**
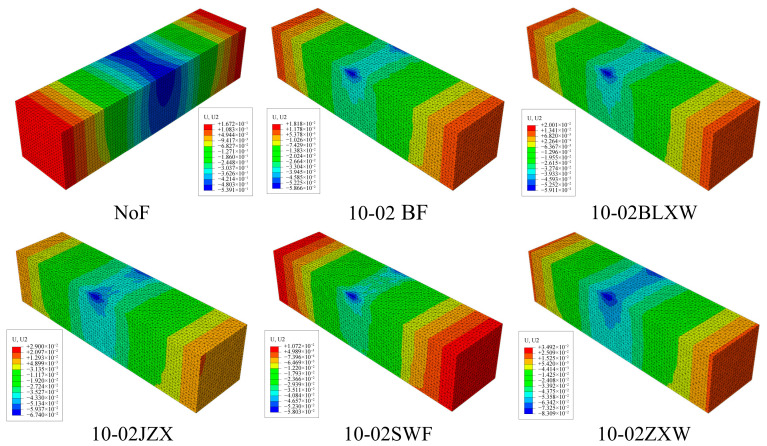
Compression displacement cloud map of asphalt mastic specimens containing different fibers.

**Figure 20:**
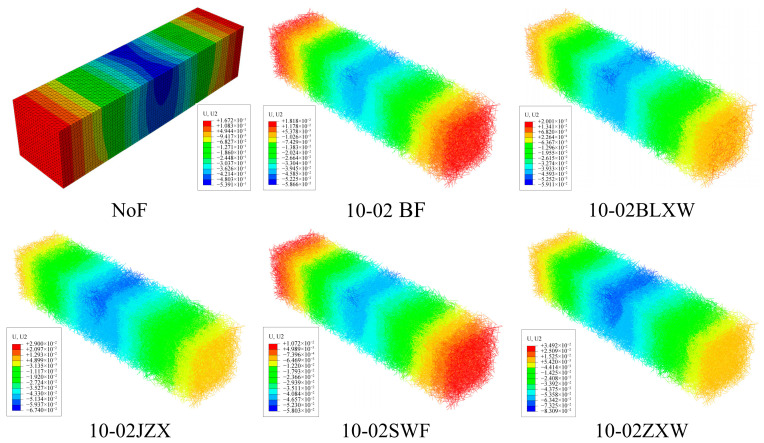
Cloud map of compression displacement of fibers in asphalt mastic specimens containing different fibers.

This paper selects BF, SWF, JZXW, BLXW, and ZXW virtual test experiments with a fiber content of 0.2% to study the influence of fiber types on the mechanical properties of asphalt mastic. Among them, all fibers have a length of 6 mm and a single fiber diameter of 0.1 mm. Figure 19 and Figure 20 show the vertical displacement distribution cloud maps of asphalt mastic specimens containing different fiber types and their internal fibers, respectively. According to Figure 19 and Figure 20, the overall displacement of the asphalt mastic specimen is significantly reduced after the addition of fibers, and the corresponding reduction rate is shown in Table 6. Among them, the decrease in ZXW fiber is the smallest (79.2%), and the decrease in SWF is the largest (93.6%). Overall, as the elastic modulus of the added fibers increases, the vertical displacement of the asphalt mastic specimens decreases and the horizontal displacement increases. The reinforcement effect of fibers on the specimens increases with the fibers’ elastic modulus.

**Figure 21:**
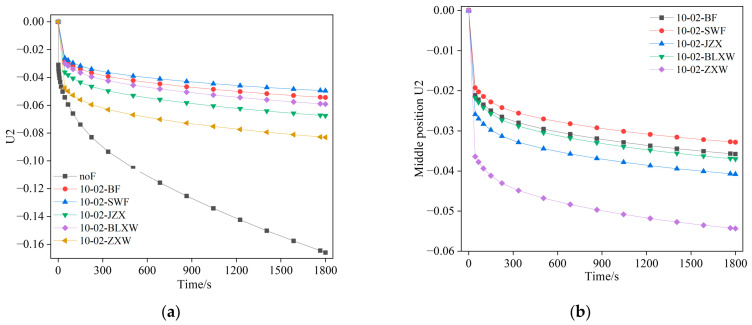
The variation in displacement of points A and B in the specimen with loading time. (**a**) Point A; (**b**) point B.

**Figure 22:**
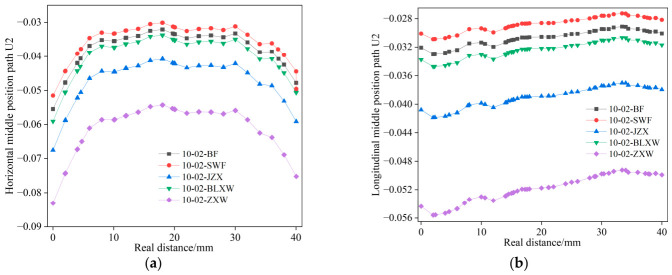
Changes in displacement of path 1 and path 2 along the true position of the specimen. (**a**) Path 1; (**b**) path 2.

Figure 21 and Figure 22, respectively, demonstrate the reinforcing effect of different fiber types on asphalt mastic, based on simulated data from different observation points. It can be seen from Figure 21 and Figure 22 that the material strength of the fiber itself determines its reinforcement effect on asphalt mastic, but it is not directly proportional.

## 4. Conclusions

This article is mainly based on the Burgers constitutive model of asphalt binder derived from low-temperature bending beam rheological tests (BBR). A three-dimensional fiber finite element model of asphalt mastic is generated in MATLAB and used to simulate a three-point bending test. The effects of fiber length, content, and type on the bending and tensile properties of asphalt mastic are analyzed. The accuracy of the three-dimensional fiber model of asphalt mastic was verified by comparing the numerical simulation results with the indoor BBR test results. The main research conclusions are as follows:

(1) Through BBR experiments, it was found that with the increase in loading time, the stiffness modulus of asphalt binder rapidly decays in the initial period, and then the growth rate tends to stabilize. Meanwhile, the stiffness modulus of the asphalt binder decreases continuously with increasing temperature, while the creep rate increases continuously.

(2) After adding fibers to the asphalt binder specimens, the rheological deformation of the asphalt mastic is significantly reduced. Fibers can have a positive reinforcing effect on asphalt mastic in fiber-composite materials. Under the same load displacement, as the length of the fiber bundle increases, the peak tensile stress decreases, and longer fiber sizes have a better reinforcing effect on asphalt binders. Adding fibers to pure asphalt binder can effectively absorb the stress in the asphalt mastic matrix and redistribute it, thereby reducing the average compressive stress between the asphalt mastic matrix and the top of the specimen.

(3) The longitudinal comparison of the enhancement effect of fiber content on asphalt mastic performance shows that, as fiber content increases, the stress field of the asphalt mastic matrix decreases, explaining the decrease in vertical deformation at the top of the fiber mastic. The effect of fiber content in the sample becomes more significant as the fiber length decreases. Similarly, the effect of fiber length becomes more pronounced as the fiber content decreases. Adding fibers to the matrix can significantly improve the rheological properties of asphalt mastic materials under compressive stress. Meanwhile, as the fiber content in the specimen increases, the peak stress of the asphalt binder gradually decreases, and the enhancement effect of fiber content on the specimen performance is significantly stronger than that of fiber length.

(4) This article investigates the influence of fiber types on the mechanical properties of asphalt mastic. When the fiber content and length are the same as the diameter of a single fiber, the decrease in ZXW is the smallest (79.2%), and the decrease in SWF is the largest (93.6%). Overall, the decrease in vertical displacement of the specimen increases with the increase in the elastic modulus of the fiber added to the asphalt mastic. The reinforcing effect of the fiber on the asphalt mastic specimen gradually increases as its own mechanical properties increase.

Future research directions include experimental validation of the predicted optimal fiber parameters through three-point bending tests on physically prepared specimens, investigation of fiber–matrix interface behavior using cohesive zone models, and multiscale modeling linking fiber-scale reinforcement to pavement-scale cracking performance.

## Figures and Tables

**Table 1:** Material properties of different fibers.

Types	Elastic Modulus (GPa)	Poisson’s Ratio	Density (g/cm^3^)	Reference Standard
BF	100	0.15	2.7	GB/T 30022-2013 [41]; ASTM D3379 [42]
SWF	200	0.15	7.5	GB/T 30022-2013 [41]
JZXW	11.8	0.15	1.38	GB/T 14344-2022 [43]
BLXW	73	0.15	2.6	ASTM D2343 [44]; GB/T 1463 [45]
ZXW	4.19	0.3	1.49	GB/T 1463 [45]

**Table 2:** Fitting results of viscoelastic parameters of 70 # matrix asphalt at three temperatures.

Model Parameters	−18 °C	−12 °C	−6 °C
E_1_	715.3	341.6	164
E_2_	580	187	63.8
$\eta_{1}$	9.298 × 10^4^	2.095 × 10^4^	5689
$\eta_{2}$	9.14 × 10^4^	6439	2455
R^2^	0.9979	0.9992	0.9996

**Table 3:** Prony series input value.

Temperature	g_1_	g_2_	$\tau_{1}$	$\tau_{2}$
−6 °C	1.40 × 10^−2^	9.86 × 10^−1^	3.96 × 10^2^	5.17 × 10^1^
−12 °C	8.56 × 10^−2^	9.14 × 10^−1^	1.97 × 10^2^	1.07 × 10^1^
−18 °C	4.41 × 10^−2^	9.56 × 10^−1^	1.54 × 10^2^	8.69 × 10^0^

**Table 4:** Variation in compressive stress with fiber content and length.

Fiber Content/%	The Compressive Stress Values of the Asphalt Mastic Model Decreased Compared to the Control Model/%		
Fiber Length/mm	0.1	0.2	0.3
6	87.17	92.3	93.7
8	88.8	94.5	95.0
10	94.1	95.8	95.9

**Table 5:** The vertical displacement of points A and B varies with the fiber content and length.

Point	Category	Fiber Content/%		
A	Fiber length/mm	0.1	0.2	0.3
	6	78.6	84.6	90.5
	8	86.8	89.1	93.4
	10	89.1	93.9	94.8
B	Fiber length/mm	0.1	0.2	0.3
	6	46.2	62.8	79.0
	8	67.3	77.5	79.4
	10	75.4	83.8	91.0

**Table 6:** The overall displacement reduction ratio of asphalt mastic specimens with different fiber types.

Types	Vertical Displacement Reduction Ratio/%
BF	89.1
BLXW	88.0
JZX	82.7
SWF	93.6
ZXW	79.2

## Data Availability

The original contributions presented in this study are included in the article. Further inquiries can be directed to the corresponding author.
